# Expression of circulating vascular endothelial growth factor-antagonizing cytokines and vascular stabilizing factors prior to and following bypass surgery in patients with moyamoya disease

**DOI:** 10.3892/etm.2014.1713

**Published:** 2014-05-14

**Authors:** JIN HE, RONG WANG, DONG ZHANG, YAN ZHANG, QIAN ZHANG, JIZONG ZHAO

**Affiliations:** Department of Neurosurgery, Beijing Tiantan Hospital, Capital Medical University, Beijing 100050, P.R. China

**Keywords:** moyamoya disease, vascular endothelial growth factor, soluble vascular endothelial growth factor receptor, angiopoietin

## Abstract

The aim of the present study was to investigate the levels of vascular endothelial growth factor (VEGF)-antagonizing cytokines and VEGF-influenced vascular stabilizing cytokines in patients with moyamoya disease (MMD) and the association with postoperative collateral vessel formation. The study population included 53 MMD patients that had undergone indirect bypass surgery and 50 healthy controls. Serum levels of VEGF, thrombospondin-1 (TSP-1), TSP-2, soluble VEGF receptor-1 (sVEGFR-1), sVEGFR-2, endostatin, angiopoietin-1 (Ang-1) and Ang-2 were measured at the baseline (preoperative) and at day seven following surgery. Postoperative collateralization assessment was conducted upon the six-month follow-up cerebral angiography. Cytokine levels were compared between patients with good or poor collateral formation. Compared with the healthy controls, MMD patients exhibited lower baseline levels of sVEGFR-1 (P<0.0001) and sVEGFR-2 (P<0.0001), but higher VEGF expression (P<0.0001). Ang-1 and Ang-2 levels did not exhibit any difference between the two groups. On day seven following surgery, MMD patients exhibited an almost unchanged sVEGFR-1 and sVEGFR-2 expression level, but upregulated expression of VEGF (P<0.0001), Ang-1 (P<0.0001) and TSP-2 (P<0.0001). The six-month follow-up angiographies revealed that 21 patients (45.65%) that had undergone the same surgical procedure achieved good collateralization. Patients with good collateral formation appeared to have lower sVEGFR-1 and sVEGFR-2 levels prior to (P=0.029 and P=0.045, respectively) and at day seven (P=0.044 and P=0.047, respectively) following bypass surgery when compared with the patients with worse collateralization. Therefore, sVEGFR-1 and sVEGFR-2 may play a role in the pathogenesis of MMD. Lower levels of sVEGFR-1 and sVEGFR-2 indicated better postoperative collateralization in the six months following indirect bypass surgery. However, Ang-1 and Ang-2 may not be specifically involved in the course of MMD.

## Introduction

Moyamoya disease (MMD) is a progressive cerebrovascular disease that is characterized by the formation of excessive collateral vessels (moyamoya vessels) at the brain base and occlusive or stenotic changes of the main cerebral arteries (1). Moyamoya vessels are hypothesized to be dilated and tortuous, perforating arteries and compensating for regional hypoxia, with thickened or thinned intima, sparse vascular smooth muscle cells (vSMCs) and fibrosis in the media (2,3). Collateral formation plays a key role in the pathogenesis of MMD (4). Currently, the surgical treatments for MMD predominantly aim at developing collateral vessels feeding from the external carotid artery system (5).

Vascular endothelial growth factor (VEGF) is a fundamental angiogenic factor in collateral vessel formation. In cerebral ischemic diseases, cerebral angiogenesis is caused by the release of VEGF (6). In addition, VEGF contributes to the course of arteriogenesis, which is the enlargement of preexisting arterioles, triggered by increased fluid shear stress (7). VEGF has been found to exhibit excessive expression in MMD patients (8–10), however, the exact role of VEGF in the pathogenesis of MMD remains unknown. Abundant VEGF does not appear to result in sufficient and persistent collateral formation in MMD. A recent genetic study revealed that among several VEGF gene polymorphisms, the CC genotype of VEGF-634 may be specifically associated with better collateral vessel formation in MMD following surgery (11). In addition to angiogenic factors, such as VEGF, vessel formation is also regulated by antiangiogenic cytokines and vascular stabilizing factors. Therefore, it is hypothesized that VEGF-antagonizing antiangiogenic factors and vascular stabilizing factors affected by VEGF may be involved in the collateralization of MMD.

The aim of the present study was to investigate the expression patterns of antagonists of VEGF and VEGF-affected vessel stabilizing factors in MMD patients preoperatively and at day seven following bypass surgery, to assess their involvement in the pathogenesis of MMD. The association between these cytokines and the six-month follow-up collateral vessel formation was also analyzed.

## Materials and methods

### Study population

The study included 53 consecutive Chinese MMD patients that had undergone indirect bypass surgery in the Stroke Center of Beijing Tiantan Hospital (Beijing, China) between March 2012 and March 2013 (Table I). All the patients were diagnosed by conventional angiography, according to the Suzuki grading method (12). The mean age of the MMD patients was 35.22±11.47 years and 32 patients (60.38%) were male. The healthy control group comprised 50 people, of which the mean age was 34.7±6.16 years and 30 individuals were male (60.00%). All the operated hemispheres were demonstrated to have decreased cerebral perfusion by perfusion computed tomography (CT) prior to surgery. The National Institutes of Health Stroke Scale score was used to assess the preoperative neurological status. Among the recruited MMD patients, 46 patients received encephalo-duro-arterio-synangiosis (EDAS) surgery, while EDAS plus a multiple burr hole procedure was applied in seven patients. Detailed surgical procedures were similar to previous descriptions (13,14). Patients were excluded from the study for the following reasons: i) Within 4 weeks of a MMD-associated intracranial hemorrhage (ICH); ii) infection within 14 days prior to admission; iii) undergoing previous neurosurgical treatment for MMD; and iv) unwilling to participate in the study.

The study was approved by the local Ethics Committee (Institutional Review Board of Beijing Tiantan Hospital, Capital Medical University) and all the procedures were conducted in accordance with the Declaration of Helsinki. All the participants and their close relatives were fully informed and provided written consent.

### Serum preparation and cytokine detection

Peripheral venous blood samples were collected from each patient on admission and at day seven following surgery (healthy controls only once). The blood samples were collected in plain serum tubes (BD Vacutainer, BD Vacutainer Systems, Plymouth, UK) and were allowed to stand for 30 min for coagulation. Serum was isolated using density gradient centrifugation for 10 min at 1,008 × g and stored at −80°C until assayed.

Quantitative measurements were performed using various protein arrays to detect the serum levels of VEGF, thrombospondin-2 (TSP-2), endostatin and angiopoietin-1 (Ang-1; VEGF, TSP-2 and Ang-1, from Fluorokine MAP Multiplex Human Angiogenesis Panel A base kit, R&D Systems, Minneapolis, MN, USA), soluble VEGF receptor-1 (sVEGFR-1) and sVEGFR-2 (sVEGFR-1 and sVEGFR-2, from Bio-Plex Pro™ Human Cancer Biomarker Panel, Bio-Rad Laboratories, Hercules, CA, USA) and Ang-2 (Milliplex MAP Human Angiogenesis/Growth Factor Magnetic Bead Panel, EMD Millipore, Billerica, MA, USA). The level of TSP-1 was determined using a commercial ELISA kit (Quantikine Human Thrombospondin-1 Immunoassay, R&D Systems, Minneapolis, MN, USA). All the procedures were conducted by strictly following the manufacturer’s instructions. Each sample was analyzed in duplicate and processed at the first freeze-thaw cycle.

### Follow-up collateral formation assessment

**Figure 1:**
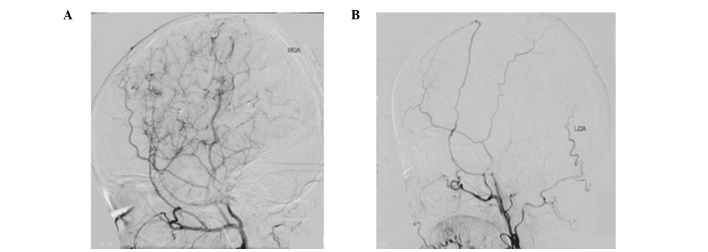
Cerebral angiographies at six months following bypass surgery in two adult cases (lateral view). Collateral vessel formation grade was based on the comparison to MCA territory. (A) Patient A received EDAS surgery and good collateralization was observed, with the collateral formation area more than one-third of the MCA territory. (B) Patient B also received EDAS surgery, but poor collateralization was observed, with almost no collateral formation in the MCA territory. EDAS, encephalo-duro-arterio-synangiosis; MCA, middle cerebral artery.

Follow-up cerebral angiography was conducted approximately six months following the bypass surgery. Newly developed collateral vessels were evaluated according to the grading method previously described by Matsushima *et al* (15). In brief, grade A represented a new collateral network covering more than one-third of the middle cerebral artery (MCA) distribution; grade B represented a network covering less than one-third of the MCA distribution, but more than two cortical branches of the MCA were supplied through the external carotid artery (ECA) system; grade C indicated that only one cortical branch of MCA was fed by the ECA system; and grade D indicated no collateral circulation. Due to the limited sample size, the patients were further divided by collateral vessel formation into good (collateral grade A) and poor (collateral grade B, C and D) groups (Fig. 1).

### Statistical analysis

Cytokine levels are presented as the mean ± standard deviation. An independent t-test was used to compare the age and cytokines levels between the MMD patients and the healthy controls. An independent t-test was also used to compare the time interval between surgery and follow-up angiography between the MMD patients with good or poor collateral formation. A paired t-test was used to compare the cytokines levels of MMD patients prior to and at day seven following surgery. A two-sided χ^2^ test was used to compare the male gender percentage between MMD patients and healthy controls, and also between MMD patients had good or poor collateral formation. Statistical analysis was conducted using Statistical Package for the Social Sciences software (SPSS; SPSS, Inc., Chicago, IL, USA), where P<0.05 or P<0.005 was considered to indicate a statistically significant difference, according to the context.

## Results

### Clinical characteristics of the MMD patients and healthy controls

Characteristics of the MMD patients and healthy controls are described in Table I. The MMD patients had no age or gender ratio discrepancy when compared with the control group. Among the participants, 22 MMD patients presented with transient ischemic attacks (TIAs), 18 patients presented with permanent stroke-related symptoms (PSRs), nine individuals presented with ICH and four patients presented with a non-specific headache. Preoperative cerebral angiography indicated that the number of hemispheres graded as Suzuki’s grade III or higher amounted to 79 (74.53%). In addition, the MCA territory was the most often involved ischemic region according to the perfusion CT scanning. The time interval between the initial onset of symptoms to surgery was less than one year in 23 patients, between one (included) and three years (not included) in 24 patients and more than three years (included) in six patients.

### Comparison of baseline cytokines levels between the MMD patients and healthy controls

Detailed immunoassay results are shown in Table II. Among the cytokines, the serum level of VEGF (P<0.0001) was higher, while the levels of sVEGFR-1 (P<0.0001) and sVEGFR-2 (P<0.0001) were significantly lower in MMD patients when compared with the healthy controls. Additional VEGF antagonists and vascular stabilizing factors, including Ang-1 and Ang-2, were not statistically different from the healthy controls.

### Comparison of cytokine levels in MMD patients at day seven and at the baseline

Detailed results are listed in Table III. At day seven following surgery, sVEGFR-1 (P=0.019) and sVEGFR-2 (P=0.249) exhibited a slight, but not significant, increase when compared with the level at the baseline. VEGF (P<0.0001), TSP-2 (P<0.0001) and Ang-1 (P<0.0001) levels all significantly increased at day seven when compared with the preoperative levels at the baseline.

### Evaluation of collateral formation and the association with sVEGFR-1 and -2 serum levels

Detailed results are listed in Table IV. Due to the limited number (n=7) of patients that received EDAS plus multiple burr hole surgery, only the patients that underwent EDAS were compared in this sector (n=46). The patients were divided according to the follow-up cerebral angiography and the good collateralization group comprised 21 cases (45.65%), while the poor group consisted of 25 cases (54.35%). Age, male gender ratio and the time interval between surgery and postoperative cerebral angiography had no discrepancy between the two groups. The cases with good collateral formation included 12 patients with TIAs, six patients with PSRs and one ICH patient. The group with poor collateralization included 10 patients with TIAs, 10 patients with PSRs and three ICH patients.

On day seven following surgery, patients with good collateralization exhibited no significant change in the levels of sVEGFR-1 (P=0.142) or sVEGFR-2 (P=0.076) when compared with the level at the baseline. Patients with poor collateralization had increased sVEGFR-1 (P=0.006) levels, while sVEGFR-2 (P=0.472) levels exhibited no significant change when compared with the baseline levels. Patients with good collateralization presented with lower levels of sVEGFR-1 and sVEGFR-2 preoperatively (P=0.029 and P=0.045, respectively) and at day seven following surgery (P=0.044 and P=0.047, respectively) when compared with the group with worse collateral formation.

## Discussion

The aim of the present study was to investigate the expression levels of VEGF-antagonizing cytokines and Ang-1 and Ang-2 in MMD patients preoperatively and at day seven following surgery. MMD patients were found to have significantly decreased levels of sVEGFR-1 and sVEGFR-2. Lower levels of sVEGFR-1 and sVEGFR-2 indicated better collateral formation at six months following indirect bypass surgery.

Collateral formation plays a key role in the pathogenesis and surgical treatment of MMD (4,5). Collateral vessel enlargement (arteriogenesis) and capillary growth (angiogenesis) are hypothesized to participate in the collateral vessel formation induced by indirect bypass surgery in MMD (16). VEGF is a specific and critical molecule during vessel formation, promoting angiogenesis and also participating in arteriogenesis (17). VEGF and VEGFR-2 binding is the initial step of angiogenesis, which breaks the vascular quiescence (18). Through VEGFR-2, VEGF promotes endothelial cells to proliferate, migrate and inhibit apoptosis (19). However, VEGFR-1 negatively regulates the angiogenic effects induced by VEGFR-2 (20). VEGFR-1 binds to VEGF and reduces the availability of VEGF to combine with VEGFR-2 (21). In addition, VEGF contributes to the course of arteriogenesis through a nitric oxide dependent pathway (7).

Although increased VEGF expression has been demonstrated in the dura matter (8), peripheral serum (9) and plasma (10) of MMD patients, the particular role of VEGF in MMD remains unclear. Since VEGF is a pivotal angiogenic factor, high concentrations in the serum of MMD patients should induce the process of neovascularization. MMD tends to develop collateral formation more easily than other cerebrovascular occlusive diseases, but the moyamoya vessels disappear at the terminal stage of MMD, depicted by the Suzuki’s grading system (12,22). Previous studies have demonstrated that VEGF deprivation impairs angiogenesis, and blocking of VEGFR-1, VEGFR-2 not only inhibits arteriogenesis but also partially decreases angiogenesis (23,24). Therefore, it is reasonable to hypothesize that VEGF antagonists (VEGF-associated antiangiogeneic factors) may play a role in the pathological course of MMD.

In the present study, levels of sVEGFR-1 and sVEGFR-2 were found to be lower in MMD patients than in healthy controls, particularly sVEGFR-2 (less than half of the controls). The expression pattern remained unchanged on day seven following indirect bypass surgery. Thus, we hypothesize that these cytokines may serve as biomarkers of MMD. sVEGFR-1 and sVEGFR-2 are soluble forms of VEGFR-1 and VEGFR-2, respectively. sVEGFR-1 potently inhibits angiogenesis by sequestering circulating VEGF, resulting in less free VEGF available to bind to VEGFR-2 (25,26). The affinity of VEGFR-1 to VEGF is >10-fold higher than VEGFR-2 (27). sVEGFR-1 is expressed and deposited by adjacent endothelial cells and may serve a role in maintaining vascular stability (28). To date, little is known about sVEGFR-2, but based on previous studies, sVEGFR-2 may also have antiangiogenic effects similar to sVEGFR-1 (26,29). Thus, decreased sVEGFR-1 and sVEGFR-2 levels, in accordance with the increased VEGF level, may facilitate collateral formation in MMD, which is distinct from other cerebrovascular occlusive diseases (22). During the course of vessel maturation, sVEGFR-1 and sVEGFR-2 recruit mural cells via a paracrine mechanism, which involves interplay in endothelial cells between VEGF/VEGFR-2 and sphingosine-1-phosphate type-1 (S1P)/S1P1 pathways, resulting in the activation of endothelial nitric oxide synthase (30). Thus, lower sVEGFR-1 and sVEGFR-2 levels may also contribute to the sparse vSMCs in the media of moyamoya vessels (2,3).

Inducing a well-developed collateral network is the aim of surgical treatments for MMD. This network is not only able to compensate for cerebral ischemia, but is also considered to prevent the recurrence of ICH (31). In the present study, MMD patients with better follow-up collateral formation had lower sVEGFR-1 and sVEGFR-2 levels prior to and at day seven following surgery when compared with the patients with worse collateralization. This observation supports the hypothesis that sVEGFR-1 and sVEGFR-2 participate in collateral vessel formation in MMD. However, this hypothesis is drawn with caution, as collateral formation is a complex process that can be manipulated by a variety of cytokines. The results of the present study are in accordance with the study by Park *et al*, where the authors demonstrated that the CC genotype of VEGF-634 contributes to better collateral formation in MMD patients following bypass surgery (11).

Endostatin, TSP-1 and TSP-2 are all VEGF-antagonizing cytokines. Endostatin inhibits VEGF-induced angiogenesis partially through blocking VEGFR-2 signaling (32). TSP-1 and TSP-2 antagonize VEGF-induced angiogenesis by CD36, CD47 and integrins, which associate with VEGFR-2 to form a platform for the integration of positive and negative signals for angiogenesis (33). Ang-1 and its receptor, Tie-2, promote vascular stabilization by recruiting pericytes and facilitate angiogenesis along with VEGF (34). Ang-2-mediated inhibition of Tie-2 signaling is required for vessel destabilization, leading to vessel growth in the presence of VEGF or to vessel regression in the absence of VEGF (35,36). However, the levels of these cytokines failed to exhibit a significant difference when compared between MMD patients and healthy controls. Considering the VEGF-antagonizing mechanism of sVEGFR-1 and sVEGFR-2, future studies on the role of VEGF in MMD should focus on the phase prior to VEGF/VEGFR-2 binding.

In the present study, the increased level of VEGF on day 7 following surgery marked an active vessel formation (19). Nakamura *et al* reported that indirect bypass surgery-induced angiogenesis can be initiated within seven days in a chronic cerebral ischemia model imitating MMD (37). Besides VEGF, angiogenesis-promoter Ang-1 and angiogenesis-blocker TSP-2 also presented increased expression 7 days after bypass surgery in our study. Thus, the study indicated that MMD patients preserve the capacity to regulate the cytokine expression that is required by vessel formation, at least partially.

There are certain limitations in the present study. Firstly, the study did not include an age-matched cerebrovascular occlusive disease control group, as usually these patients are elderly. This impaired the strength of the observations. Secondly, blood samples and cerebral angiographic assessment were not analyzed at multiple time points during the postoperative follow-up. Thus, how the levels of cytokines change and whether the changes are consistent with the progression of Suzuki’s grading remain unknown. Thirdly, the sample size was small, therefore, the data should be interpreted with caution. In the future, the observations require further validation by conducting studies with larger cohorts.

In conclusion, the present study indicated that antiangiogenic cytokines, including sVEGFR-1 and sVEGFR-2, may be involved in collateral formation in MMD. In addition, the preoperative and day seven postoperative serum levels indicated an association with the six-month follow-up collateralization status following indirect bypass surgery. However, the results indicated that Ang-1 and Ang-2 may not be specifically involved in the course of MMD.

## Figures and Tables

**Table I:** Clinical characteristics of the MMD patients and healthy controls.

Characteristics	Healthy controls (n=50)	MMD patients (n=53)	P-value
Age (years; mean ± SD)	34.7±6.16	35.22±11.47	0.767^a^
Male gender (%)	60	60.38	1.000^b^
Cerebral angiography Suzuki’s grading (hemispheres, n)			
II	-	26	
III	-	31	
IV	-	37	
V	-	9	
VI	-	3	
Location of ischemic region (hemispheres, n)			
ACA territory	-	55	
MCA territory	-	87	
PCA territory	-	34	
Categories by chief symptom (n)			
TIAs	-	22	
PSRs	-	18	
ICH	-	9	
Non-specific	-	4	
NIHSS on admission (mean ± SD)	-	1.11±2.19	
Type of surgery (n)			
EDAS surgery	-	46	
EDAS surgery plus multiple burr hole	-	7	

P<0.05 was considered to indicate a statistically significant difference, as determined by an ^a^independent t-test and ^b^two-sided χ^2^ test. MMD, moyamoya disease; ACA, anterior cerebral artery; MCA, middle cerebral artery; PCA, posterior cerebral artery; EDAS, encephalo-duro-arterio-synangiosis; TIAs, transient ischemic attacks; PSRs, permanent stroke-related symptoms; ICH, intracranial hemorrhage; NIHSS, National Institutes of Health Stroke Scale.

**Table II:** Comparison of serum cytokines levels between the healthy controls and preoperative MMD patients.

Cytokines	Healthy controls (pg/ml; n=50)	MMD prior to surgery (pg/ml; n=53)	P-value^a^
VEGF	89.47±68.30	178.74±49.95	<0.0001
sVEGFR-1	108.80±33.47	71.15±18.00	<0.0001
sVEGFR-2	3009.10±1209.83	1401.59±1163.58	<0.0001
TSP-1	234.96±69.29	215.62±75.96	0.366
TSP-2	12815.40±5209.16	8880.07±5414.17	0.014
Endostatin	42051.19±12964.90	48776.13±11708.16	0.063
Ang-1	33612.53±11479.40	26821.96±7471.17	0.026
Ang-2	1280.92±630.99	1235.68±610.21	0.801

a P<0.005 was considered to indicate a statistically significant difference, as determined by an independent t-test. Results are expressed as the mean ± SD.

MMD, moyamoya disease; VEGF, vascular endothelial growth factor; sVEGFR, soluble vascular endothelial growth factor receptor; TSP, thrombospondin; Ang, angiopoietin.

**Table III:** Comparison of cytokines levels between MMD patients at the baseline and at day seven following surgery.

Cytokines	Baseline (pg/ml; n=50)	Day 7 following surgery (pg/ml; n=53)	P-value^a^
VEGF	178.74±49.95	361.00±199.34	<0.0001
sVEGFR-1	71.15±18.00	89.00±31.88	0.019
sVEGFR-2	1401.59±1163.58	1792.70±1249.95	0.249
Endostatin	48776.13±11708.16	46332.85±14592.42	0.221
TSP-1	215.62±75.96	187.29±66.83	0.043
TSP-2	8880.07±5414.17	12605.01±7990.00	<0.0001
Ang-1	26821.96±7471.17	36194.26±14310.27	<0.0001
Ang-2	1235.68±610.21	1652.76±1274.39	0.030

a P<0.005 was considered to indicate a statistically significant difference, as determined by a paired t-test. Results are expressed as the mean ± SD.

MMD, moyamoya disease; VEGF, vascular endothelial growth factor; sVEGFR, soluble vascular endothelial growth factor receptor; TSP, thrombospondin; Ang, angiopoietin.

**Table IV:** Clinical characteristics of the MMD patients according to collateral formation grading.

Characteristics	Good collateralization group (n=21)	Poor collateralization group (n=25)	P-value
Age (years; mean ± SD)	34.86±10.35	36.00±13.41	0.798^a^
Male gender (%)	57.14	62.50	1.000^b^
Interval between surgery and postoperative cerebral angiography (months)	5.40±0.54	6.35±0.47	0.166^a^
Categories by chief symptom (n)			
TIAs	12	10	
PSRs	6	10	
ICH	1	3	
Non-specific	2	2	
Preoperative serum level (pg/ml; mean ± SD)			
sVEGFR-1	63.61±12.75	77.74±19.66	0.029^a^
sVEGFR-2	951.42±504.27	1795.48±1429.18	0.035^a^
Postoperative serum level (pg/ml; mean ± SD)			
sVEGFR-1	76.58±28.30	99.87±31.64	0.044^a^
sVEGFR-2	1328.57±731.30	2148.94±1355.09	0.047^a^

P<0.05 was considered to indicate a statistically significant difference, as determined by an ^a^independent t-test and ^b^two-sided χ^2^ test. MMD, moyamoya disease; TIAs, transient ischemic attacks; PSRs, permanent stroke related symptoms; ICH, intracranial hemorrhage; sVEGFR, soluble vascular endothelial growth factor receptor.
